# Supplementary material for: Content-rich biological network constructed by mining PubMed abstracts
Source: BMC Bioinformatics. 2004 Oct 8;5:147. doi: 10.1186/1471-2105-5-147 (PMC528731; doi:10.1186/1471-2105-5-147)
Supplement: Additional File 2 — The original results of the above study (non-essential files are deleted to keep the file size under the limit set by BMC bioinformatics). [file 1471-2105-5-147-S2.bz2 › chilibotAdditionalFile2/dip40/291ID1372994E1122/html/WEE1_CDC2.html]

 


 **WEE1** and **CDC2** 
  
Found 181 abstracts in PubMed, retrieved 40.  
 

 What does Google say? 
 PDF only 
| .edu only 

---

**Interactive relationship** (e.g. stimulation, inhibition, etc)

**Inhibitory relationship**- In eukaryotic cells, the  **Wee1**  protein kinase phosphorylates and inhibits  **Cdc2** , thereby creating an interphase of the cell cycle.  Ref: 12006499 EMBO J, 2002
- The inhibition of p34  **cdc2**  kinase activity by apigenin treatment correlated with increasing levels of p34  **cdc2**  phosphorylation at Tyr15, a site in the p34  **cdc2**  kinase that undergoes inhibitory phosphorylation by  **Wee1**  kinase.  Ref: 11807956 Mol Carcinog, 2002
- Of the differentially expressed genes encoding proteins regulating the G 2 to M transition, chk1,  **wee1** , and GADD45 are known to inhibit  **cdc2**  kinase activity.  Ref: 11861824 J Virol, 2002
- The  **WEE1**  protein kinase carries out the inhibitory phosphorylation of  **CDC2**  on tyrosine 15 Tyr15, which is required for activation of the G 2 phase checkpoint in response to DNA damage.  Ref: 11839095 Radiat Res, 2002
- The  **Wee1**  protein kinase negatively regulates entry into mitosis by mediating the inhibitory tyrosine phosphorylation of  **Cdc2**  cyclin B kinase.  Ref: 11298745 Eur J Biochem, 2001
- **Wee1**  kinases catalyze inhibitory phosphorylation of the mitotic regulator Cdk1  [ **CDC2** ] , preventing mitosis during S phase and delaying it in response to DNA damage or developmental signals during G2.  Ref: 12072468 Genetics, 2002
- **Cdc2**  regulation also involves positive feedback, with active  **Cdc2**  cyclin B stimulating its activator Cdc25 refs 5 7 and inactivating its inhibitors  **Wee1**  and Myt1 refs 8 11.  Ref: 12629549 Nat Cell Biol, 2003
- The activity of  **cdc2**  associated kinase was decreased in both cell lines, which was accompanied by induction of  **Wee1** .  Ref: 12406077 Br J Haematol, 2002
- In particular, augmentation of CDK  [ **CDC2** ]  inhibitors p21cip1 and p27kip1 and  **Wee1**  kinase and diminution of CDC25C phosphatase coincided with induced Tyr 15 phosphorylation and inhibition of  **CDC2** .  Ref: 12669309 Mol Carcinog, 2003
- As other Cdk  [ **CDC2** ]  inhibitors, such as roscovitine and Myt1, did not act like  **Wee1**  to accelerate apoptosis, we propose that  **Wee1**  Crk complexes signal in a novel apoptotic pathway, which may be unrelated to  **Wee1**  s role as a cell cycle regulator.  Ref: 11134069 J Cell Biol, 2000
- We show that BRCA1 regulates the expression of both  **Wee1**  kinase, an inhibitor of  **Cdc2**  cyclin B kinase, and the 14 3 3 family of proteins that sequesters phosphorylated Cdc25C and  **Cdc2**  cyclin B kinase in the cytoplasm.  Ref: 11836499 Nat Genet, 2002
- In many cells the timing of entry into mitosis is controlled by the balance between the activity of inhibitory  **Wee1**  related kinases Swe1p in budding yeast and the opposing effect of Cdc25 related phosphatases Mih1p in budding yeast that act on the cyclin dependent kinase  **Cdc2**  Cdc28p in budding yeast.  Ref: 11283616 Nat Cell Biol, 2001
**Neutral relationship**- **Wee1**  inactivates the  **Cdc2**  cyclin B complex during interphase by phosphorylating  **Cdc2**  on Tyr 15.  Ref: 11251070 Mol Biol Cell, 2001
- Little is known about the genes that regulate cyclinB  **Cdc2**  complexes at the G2 M transition of the plant cell cycle although in yeast and animals cdc25 and  **wee1**  are central regulators of  **cdc2** .  Ref: 12111237 Planta, 2002
- We show that in G1 arrested orp1 4 cells,  **Wee1**  phosphorylates and inactivates  **Cdc2** .  Ref: 12186947 J Cell Sci, 2002
- Human  **Wee1**  is a tyrosine specific protein kinase that phosphorylates  **Cdc2**  on tyrosine 15.  Ref: 11751453 Cell Growth Differ, 2001
- PP2A is known to interact genetically in fission yeast with the  **Wee1**  kinase and Cdc25 phosphatase that act on Tyr15 of  **Cdc2** .  Ref: 11531413 Virology, 2001
- Ubiquitination of Plk1 by Chfr delays the activation of the Cdc25C phosphatase and the inactivation of the  **Wee1**  kinase, leading to a delay in  **Cdc2**  activation.  Ref: 11807090 J Cell Biol, 2002
- M phase promoting factor is a complex of  **cdc2**  and cyclin B that is regulated positively by cdc25 phosphatase and negatively by  **wee1**  kinase.  Ref: 11500387 EMBO J, 2001
- Protein abundance for total  **Cdc2** ,  **Cdc2**  phosphorylated at the threonine 161 residue, Wee 1  [ **WEE1** ] , Myt 1, Chk 1, and 14 3 3sigma was not affected by treatment with ACNU in either cell line.  Ref: 12222840 J Neurooncol, 2002
- Last, the concentration of cdc25C is greater than that of  **wee1**  and the concentration of each is greater than that of p34  **cdc2**  in both meiotically incompetent and competent oocytes.  Ref: 11090427 Biol Reprod, 2000
- These data suggest that  **Wee1**  functions in normal postmitotic neurons, but is altered in AD so as to promote activation of  **Cdc2**  cyclin B1.  Ref: 12214061 J Alzheimers Dis< MedlineTA>J Alzheimers Dis, 2000
- Degradation of Tome 1 during G1 allows for wee 1  [ **WEE1** ]  accumulation during interphase, thereby providing a critical link between the APC and SCF pathways in regulation of cdk1  [ **CDC2** ]  cyclin B activity and thus mitotic entry and exit.  Ref: 12679038 Cell, 2003
- Lengthening of the cell cycle at the MBT is caused by increased Tyr15 phosphorylation of  **Cdc2**  resulting from degradation of the maternal phosphatase Cdc25A and continued expression of maternal  **Wee1** .  Ref: 11444050 Novartis Found Symp, 2001
- The Wee  [ **WEE1** ]  kinases block entry into mitosis by phosphorylating and inhibiting the activity of the mitotic cyclin dependent kinase, Cdk1  [ **CDC2** ] .  Ref: 12217326 Dev Biol, 2002

**Non-interactive relationship** (e.g. studied together, co-existance, homology, etc.)

- Identification and characterization of human Wee1B, a new member of the  **Wee1**  family of Cdk  [ **CDC2** ]  inhibitory kinases.  Ref: 11029659 Genes Cells, 2000
- These findings suggest that TGF beta 1 induced G2 arrest in Hep3B at least in part through stabilization of  **Wee1**  and subsequent increase in Tyr 15 phosphorylation and inhibition of  **CDC2** .  Ref: 12669309 Mol Carcinog, 2003
- Because  **Wee1**  kinase is crucial in maintaining G 2 arrest through its inhibitory phosphorylation of  **Cdc2** , we developed a high throughput mass screening assay and used it to screen chemical library for  **Wee1**  inhibitors.  Ref: 11719452 Cancer Res, 2001
- In antigen specific Th1 cells stimulated by antigen, transactivation of the c fos and  **wee1**  kinase genes occurred sequentially at G 1 S, and the substrate of  **wee1**  kinase,  **cdc2**  Tyr15, was subsequently phosphorylated at late G 1 S.  Ref: 11500387 EMBO J, 2001
- Here, we present the identification of Drosophila Myt1 dMyt1 kinase and examine the relationship of Myt1 and  **Wee1**  activities in the context of  **cdc2**  phosphorylation.  Ref: 11882391 Cell Signal, 2002
- Our results suggest that the PDP induced G2 M arrest in HT 29 cells was through the intracellular events including a inhibition of normal mitotic spindle formation, b elevation of cyclin B1  **cdc2**  kinase activity, c concomitant increases in cdc 25 A phosphatase and cdk  [ **CDC2** ]  7 kinase activity, and d down regulation of the wee 1  [ **WEE1** ]  protein expression.  Ref: 12049842 Toxicology, 2002
- Acquisition of meiotic competence in mouse oocytes absolute amounts of p34  **cdc2** , cyclin B1, cdc25C, and  **wee1**  in meiotically incompetent and competent oocytes.  Ref: 11090427 Biol Reprod, 2000
- This phenotype was reduced by accelerating mitotic commitment through genetic inhibition of  **Wee1**  or activation of either Cdc25 or  **Cdc2** .  Ref: 12065422 EMBO J, 2002
- These findings suggest that the G2 arrest produced by heavy ion beams, such as the carbon ion irradiation used in this study, might be associated with the overexpression of the  **Wee1**  protein and of  **Cdc2**  phosphorylation regulated by  **Wee1** .  Ref: 12787730 Cancer Detect Prev, 2003
- To investigate the mechanism coupling growth protein synthesis with cell division, we examined the relationship between the tyrosine kinase  **Wee1**  that inhibits  **Cdc2**  Cdc13 mitosis inducing kinase by phosphorylating it, and protein synthesis inhibition in fission yeast.  Ref: 11119724 FEBS Lett, 2000
- Ectopic expression of the Drosophila Cdk1  [ **CDC2** ]  inhibitory kinases,  **Wee1**  and Myt1, interferes with the second mitotic wave and disrupts pattern formation during eye development.  Ref: 12072468 Genetics, 2002
- In cycling cells the timing of  **cdc2**  activation and onset of mitosis are determined by the  **Wee1**  tyrosine kinase.  Ref: 12214061 J Alzheimers Dis< MedlineTA>J Alzheimers Dis, 2002
- This phenotype is observed in  **cdc2**  3w  **wee1**  50 cells at high temperature and is suppressed by a single recessive mutant, mcs3 12.  Ref: 11085271 Mol Gen Genet, 2000
- Tome 1 associates with Skp 1 and is required for degradation of the cdk1  [ **CDC2** ]  inhibitory tyrosine kinase  **wee1** .  Ref: 12679038 Cell, 2003
- For this we studied the effect of CA4P on selected markers of apoptosis caspases 9 and 3, poly ADP ribose polymerase PARP, bcl 2, and bax and G2 M protein regulators p53, MDM 4 3 3sigma, GADD45,  **cdc2** , cdc25, chk1,  **wee1** , p21, and cyclin B1.  Ref: 12171907 Clin Cancer Res, 2002
- We report measurements of the absolute mass of p34  **cdc2**  and cyclin B1, as well as the two proximal regulators of MPF, namely cdc25C and  **wee1** , in meiotically incompetent and competent mouse oocytes.  Ref: 11090427 Biol Reprod, 2000
- The identification of this new member of the  **Wee1**  family suggests that inhibition of Cdk1  [ **CDC2** ]  is mediated at multiple levels in mammals.  Ref: 11029659 Genes Cells, 2000
- The enzymatic activities of cyclins D1, E, A, Cdk1  [ **CDC2** ] , Cdk4, Cdk6, Cdk7, and  **Wee1**  were measured using in vitro kinase assays.  Ref: 12601350 Hepatology, 2003
- We find that the numbers of molecules of p34  **cdc2** , cyclin B1, cdc25C, and  **wee1**  in meiotically incompetent oocytes are 1.4 x 10 1.3 x 10 4.6 x 10 5.  Ref: 11090427 Biol Reprod, 2000
- Although the levels of  **Wee1**  and  **Cdc2**  protein were increased after exposure to either beam,  **Wee1**  protein levels were influenced more by carbon ion beam irradiation than by gamma rays.  Ref: 12787730 Cancer Detect Prev, 2003
- Entry into mitosis is regulated by inhibitory phosphorylation of  **cdc2**  cyclin B, and these phosphorylations can be mediated by the Wee  [ **WEE1** ]  kinase family.  Ref: 11882391 Cell Signal, 2002
- Under prolonged expression of the c fos gene the amount of  **wee1**  kinase was increased and its target  **cdc2**  molecule was constitutively phosphorylated on its tyrosine residue, where Th1 cells went into aberrant mitosis.  Ref: 11500387 EMBO J, 2001
- Mcs3 acts independently of the  **Wee1**  kinase and Cdc25 phosphatase, two major regulators of  **Cdc2** .  Ref: 11085271 Mol Gen Genet, 2000
- Exposure of WSU CLL cells to 4 and 5 nM CA4P was associated with overproduction of total p53 and no dramatic change in MDM 4 3 3sigma, GADD45, the cyclin dependent kinase  **cdc2** , its inhibitory phosphorylation, the  **cdc2**  inhibitory kinase  **wee1** , chk1, or cdc25 hyperphosphorylation.  Ref: 12171907 Clin Cancer Res, 2002
- In this study, we investigated the expression of  **Wee1**  and  **Cdc2**  protein levels in order to analyze the G2 arrest caused by carbon ion beam irradiation.  Ref: 12787730 Cancer Detect Prev, 2003
- Surprisingly however, a reduction in the phosphorylation of  **cdc2**  on the tyrosine 15 Tyr 15 residue was only observed when both dMyt1 and dWee1 expression was reduced via RNAi and not by  **Wee1**  alone.  Ref: 11882391 Cell Signal, 2002
